# Distinguishing Anesthetized from Awake State in Patients: A New Approach Using One Second Segments of Raw EEG

**DOI:** 10.3389/fnhum.2018.00040

**Published:** 2018-02-20

**Authors:** Bjørn E. Juel, Luis Romundstad, Frode Kolstad, Johan F. Storm, Pål G. Larsson

**Affiliations:** ^1^Department of Molecular Medicine, Brain Signaling, Institute of Basic Medical Science, University of Oslo, Oslo, Norway; ^2^Department of Anesthesiology, Rikshospitalet, Oslo University Hospital, Oslo, Norway; ^3^Department of Neurosurgery, Rikshospitalet, Oslo University Hospital, Oslo, Norway

**Keywords:** monitoring general anesthesia, directed transfer function (DTF), electroencephalography (EEG), consciousness, general anesthesia

## Abstract

**Objective:** The objective of this study was to test whether properties of 1-s segments of spontaneous scalp EEG activity can be used to automatically distinguish the awake state from the anesthetized state in patients undergoing general propofol anesthesia.

**Methods:** Twenty five channel EEG was recorded from 10 patients undergoing general intravenous propofol anesthesia with remifentanil during anterior cervical discectomy and fusion. From this, we extracted properties of the EEG by applying the Directed Transfer Function (DTF) directly to every 1-s segment of the raw EEG signal. The extracted properties were used to develop a data-driven classification algorithm to categorize patients as “anesthetized” or “awake” for every 1-s segment of raw EEG.

**Results:** The properties of the EEG signal were significantly different in the awake and anesthetized states for at least 8 of the 25 channels (*p* < 0.05, Bonferroni corrected Wilcoxon rank-sum tests). Using these differences, our algorithms achieved classification accuracies of 95.9%.

**Conclusion:** Properties of the DTF calculated from 1-s segments of raw EEG can be used to reliably classify whether the patients undergoing general anesthesia with propofol and remifentanil were awake or anesthetized.

**Significance:** This method may be useful for developing automatic real-time monitors of anesthesia.

## Introduction

Monitoring the level of consciousness in patients undergoing general anesthesia is important in clinical medicine. Every year, about 26,000 patients experience unintended intra-operative awareness during general anesthesia in the US alone (Sebel et al., 2004; Ghoneim et al., 2009). To reduce this problem, devices designed for automated monitoring of patients' conscious state (i.e., whether they are fully anesthetized or awake) are in clinical use (Bischoff and Rundshagen, 2011; Punjasawadwong et al., 2014). However, the reliability and validity of the existing monitors are debated (Myles et al., 2004; Avidan et al., 2008; Schnakers et al., 2008; Mashour et al., 2012; Goddard and Smith, 2013), and there is still a need for improved clinically viable tools for monitoring depth as well as quality of general anesthesia (Kreuzer, 2017).

The search for such tools should be guided by scientific findings related to changes in brain activity induced by loss of consciousness in general, and general anesthesia in particular. Over the past decades, studies have revealed many reproducible changes in electroencephalography (EEG) activity related to anesthesia-induced loss of consciousness, such as changes in frontal low frequency power and coherence in EEG signals (Mhuircheartaigh et al., 2013; Purdon et al., 2013) and a partial reduction in functional and effective connectivity (Alkire, 2008; Hudetz, 2012), as well as signal complexity of perturbed and spontaneous EEG (Casali et al., 2013; Schartner et al., 2015). Altered cortico-cortical connectivity between frontal and parietal regions has been reported to underlie spectral EEG changes during propofol-induced loss of consciousness (Boly et al., 2012). Indeed, changes in fronto-parietal connectivity upon loss of consciousness have been reported across a range of anesthetics with distinct molecular and neurophysiologic effects (Lee et al., 2009, 2013; Hudetz and Mashour, 2016), and also in sleep (Kaminski et al., 1995; Gennaro et al., 2004) and disorders of consciousness (Boly et al., 2011; Di Perri et al., 2014). However, there is also evidence speaking against the hypothesis that prefrontal cortical areas and their connections to other parts of the cortex are crucial for consciousness, and this issue is currently debated (Koch et al., 2016; Boly et al., 2017; Storm et al., 2017).

Nevertheless, there is substantial evidence that there are several changes in functional and effective connectivity during general anesthesia (Alkire, 2008; Hudetz, 2012), and for the practical purpose of finding an EEG marker for monitoring level of consciousness under anesthesia, measures quantifying brain connectivity have proven to be among the most useful (Höller et al., 2014). Unfortunately, most of the promising measures that have been used experimentally to distinguish states of consciousness require extensive data processing, long recordings, repeated measurements, or combinations of these. Although these requirements may often be met in basic research, such time-consuming steps are problematic for clinical applications and make the measures unsuitable for real-time monitoring of the conscious state during anesthesia in patients.

Here, we investigated whether properties of short segments of spontaneous, raw scalp EEG could be sufficient for distinguishing the anesthetized state from the awake state in patients. To this end, we applied a mathematical procedure called the *Directed Transfer Function* (DTF) (Kaminski and Blinowska, 1991) to one-second (1-s) segments of raw EEG data collected from patients undergoing general anesthesia. The DTF is a measure of apparent “effective connectivity” in the Granger causality family. It quantifies the apparent relevant influence of one time-series on another, at a specific frequency, given a multivariate autoregressive (MVAR) model (Greenblatt et al., 2012). For an in depth mathematical description of the DTF, see for example (Kaminski and Blinowska, 1991; Kaminski et al., 2001; Gennaro et al., 2004; Florin et al., 2011).

The DTF has previously been used to measure changes in directed connectivity when healthy volunteers fall asleep, and to characterize the level of awareness in patients suffering from disorders of consciousness (DOC) (Kaminski et al., 1995; Gennaro et al., 2004; Bertini et al., 2009; Höller et al., 2014). However, to our knowledge, there are no previous studies investigating how properties of the DTF changes when patients undergo general anesthesia.

We aimed to test whether properties of the DTF, calculated from 1 s segments of raw EEG, were sufficient to distinguish between the awake and anesthetized states in a group of patients undergoing propofol anesthesia during surgery.

We hypothesized that the analysis would reveal significant changes in the DTF properties when patients become anesthetized, and that the observed changes could be used to distinguish between awake and anesthetized states. The main results of this study have previously been presented in abstract form at meetings (e.g., Juel et al., 2016).

## Material and methods

### Study design

This was a single-center observational study of patients undergoing general propofol anesthesia with remifentanil. The patients included in the study were scheduled for anterior cervical discectomy and fusion, and the surgery was performed under total intravenous general anesthesia at Oslo University Hospital, Rikshospitalet between August and December 2013. The study was approved by the Regional Committee for Research Ethics (case number 2012/2014), and all patients included in the study signed a written consent form after oral and written information.

### Inclusion and exclusion criteria

The patients included in this study were (1) American Society of Anesthesia I–III patients (ASA Physical Status Classification System. American Society of Anesthesiologists; https://www.asahq.org/resources/clinical-information/asa-physical-status-classification-system) (2) between 18 and 83 years old, and (3) seen as otherwise healthy based on a complete health examination. Patients were excluded if they had known hypersensitivity to any of the anesthetic drugs, soy oil or egg allergy, liver or renal disease affecting drug pharmacodynamics, heart or lung disease causing physical limitations (unable to climb two stairs without rest), any abuse of drugs and alcohol causing impaired general health, organ damage, or neurological or psychiatric disease.

### Anesthetic management

The premedication consisted of oral paracetamol (Paracet®, Weifa, Oslo, Norway) 1.5 g, midazolam (Dormicum®, Basel, Switzerland) 3.75–7.5 mg for sedation, and oxycodone sustained release tablet (opioid analgesic; OxyContin®, Dublin, Ireland) 10 mg. The drugs used for anesthesia were propofol (general anesthetic) 20 mg/ml (Propolipid®, Fresenius Kabi, Uppsala, Sweden) and remifentanil (a potent, short-acting synthetic opioid analgesic) 50 μg/ml (Ultiva®, GlaxoSmithKline, Parma, Italy) administered by computer controlled infusion pumps (B Braun Perfusor Space®, Melsungen, Germany) programmed to achieve brain concentrations of the anesthetic drugs resulting in anesthesia and analgesia sufficient for surgery. Before induction of anesthesia, all patients received a 5 ml/kg IV infusion of Ringer's-Acetate counteracting hypotension during induction. When the anesthesia started, the patients were given 3–5 ml lidocaine 10 mg/ml IV to avoid injection pain from propofol. Before the patients stopped verbally communicating because of the anesthesia, they were pre-oxygenated with 100% oxygen, and as soon as they stopped spontaneous breathing due to respiratory depression from the remifentanil/propofol combination, manual ventilation with 100% oxygen was started.

The brain is the target and effect-site organ for propofol and remifentanil, and the target controlled infusion (TCI) program used for propofol was the Schnider model (Schnider et al., 1998) for effect-site TCI in μg/ml. For remifentanil the Minto model (Minto et al., 1997) was used for effect site TCI in ng/ml. The calculated effect-site/brain concentration of propofol necessary for surgical anesthesia varied between 2.5 and 5 μg/ml and the brain concentration range of remifentanil was 3–8 ng/ml. The highest effect-site concentrations were necessary during the intubation procedure, and at the start of surgery. To facilitate endotracheal intubation, a single IV dose, 2 mg/kg, of the non-depolarizing muscle relaxant cisatracurium 2 mg/ml (Nimbex®, GlaxoSmithKline, Oslo, Norway) was given. As soon as correct placement of the endotracheal tube was verified, mechanical ventilation with 40% oxygen was started. Nitric oxide was not used. All the patients received local anesthetic infiltration with 5 ml 5% bupivacaine in the area of the skin incision.

### Clinical assessment of consciousness

In the present study, the state of the patient was continuously assessed clinically by the anesthesiologist, using standard tools and practices. The Modified Observer's Assessment of Alertness/Sedation Scale (MOAAS) (Chernik et al., 1990) was used to measure the depth of sedation, until loss of verbal contact and loss of response was reached. The MOAAS assessment was employed by the anesthesiologist maintaining verbal communication with the patient during propofol administration, and the patients were considered anesthetized and unconscious when they could no longer maintain verbal communication or respond to their name being called (MOAAS level 2). When the patient could no longer communicate, the MOAAS assessment was discontinued, until the patient was about to wake up again. The time points of propofol administration, loss of consciousness as assessed clinically (i.e., corresponding to loss of verbal contact and behavioral response), and return of verbal communication after anesthesia were recorded immediately. The depth of anesthesia was continuously monitored clinically by observing the heart rate, blood pressure, sweating, tear production, eye and eyelid reflexes, pupil size and symmetry, and any limb movements with standard clinical equipment. Furthermore, the raw EEG, especially the recordings from the frontal electrodes, were observed providing information regarding the depth of anesthesia. Blood pressure was measured noninvasively with an oscillometric blood pressure monitor.

### EEG methods

**Figure 1:**
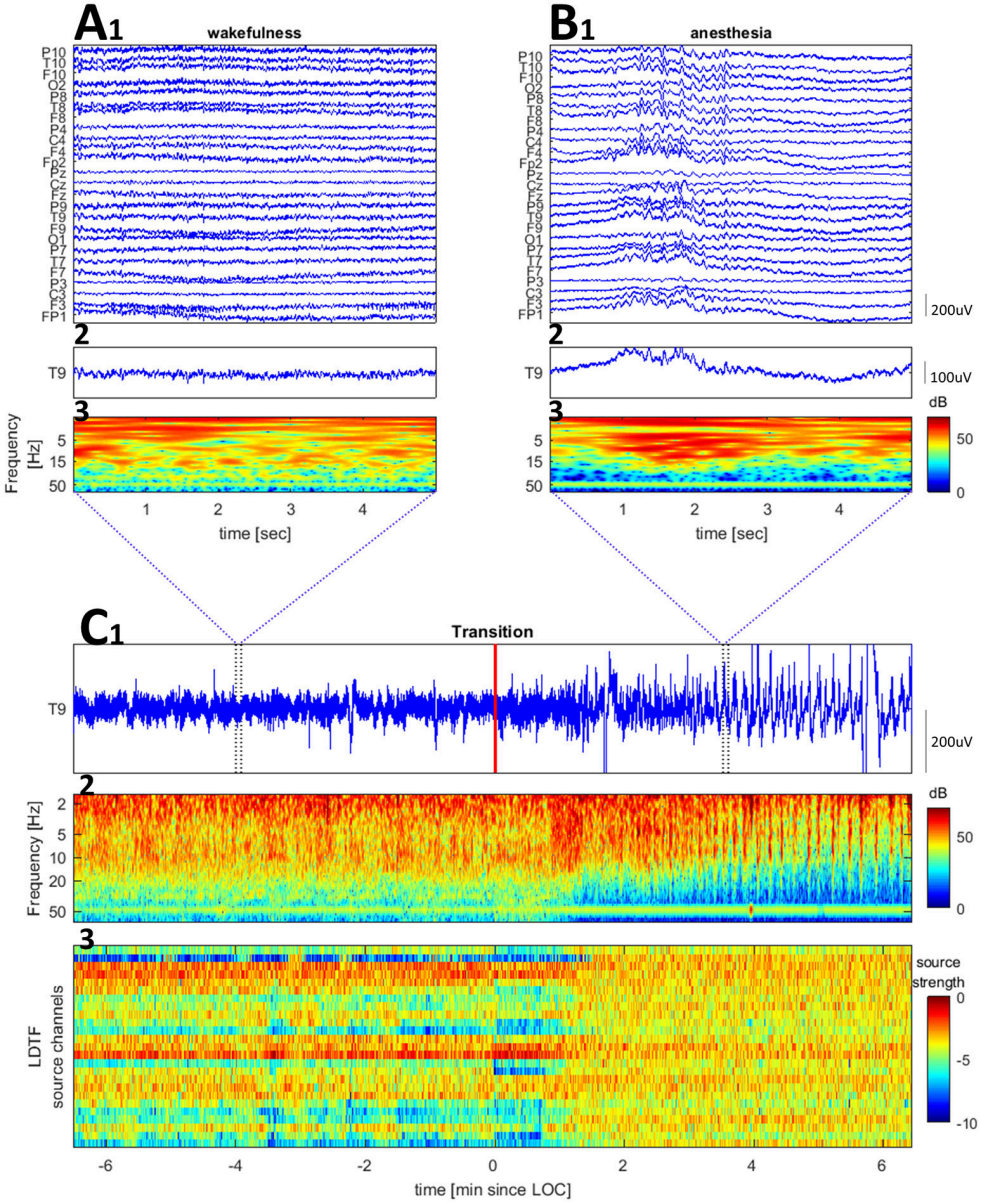
Examples of raw EEG and time-frequency plots for a channel (Cz of patient 6) and the LDTF timecourse across the transition from wake to anesthesia. **(A,B)** Show zoomed segments of raw EEG samples and time-frequency plot taken from the regions marked by blue vertical bars in **(C)**. The wavelet transform used 2 s windows, with 68 wavelets (covering 2–70 Hz) each with 12 cycles within the window. **(C)** Shows a raw EEG (top), time-frequency plot of wavelet transformed EEG data (middle), and LDTF source strength timecourse (bottom) accross the wakefulness to anesthesia transition (the red vertical line marks the time at which the anesthesiologist indicated the patient was anesthetized).

Twenty five channel EEG was recorded during the surgical procedure. The electrodes were placed in accordance with the 10–20 system with additional low row electrodes (F9, F10, T9, T10, P9, P10). The reference electrode was placed at CP1 (10–10 system). The signal was recorded through an EEG amplifier (Pleasanton, California, USA) and digitized at 512 samples per second. The signal was bandpass filtered between 0.5 and 70 Hz upon acquisition. Figure 1 shows examples of raw and wavelet transformed EEG data.

For all patients, the EEG recordings were split into three segments: before anesthesia, during anesthesia, and after anesthesia. However, due to technical difficulties, the recording of patient #2 started only after the anesthetics were administered. In the analysis, patients were considered “awake” before administration of anesthesia and after waking up from the anesthesia with regained verbal communication. These EEG segments were pooled to make up the “awake” state data. Similarly, the EEG recordings gathered while the patient was considered to be under stable general anesthesia (from 10 min after starting the administration of the anesthetics until 10 min before the patients woke up and was able to communicate verbally) were pooled to make up the “anesthetized” state data.

### Data processing

The raw EEG data was inspected using BESA Research software (version 6.0; BESA GmbH, 82166 Gräfelfing Germany; http://www.besa.de/), and time points for the administration of anesthesia, loss of verbal contact, and return of verbal communication were extracted. The data were then loaded into Matlab R2015a using the Biosys toolbox as implemented in EEGlab (Delorme and Makeig, 2004), and the resulting data was processed using in-house scripts. Specifically, the “DTF” function in the eConnectome toolbox (He et al., 2011) was employed to calculate the DTF for every 1-s segment of the raw data, without any filtering or artifact removal. Filtering and artifact removal was deliberately avoided to simulate a clinical setting, and comply with the demands of clinically viable real-time monitors of anesthesia.

In this study, we applied analyses in a similar way to Schumacher et al. (2015), calculating the DTF in the alpha band (8–12 Hz) from 1-s segments of EEG and using a relatively low MVAR model order, *O* = 8. (These were the only parameters applied for the calculation of the DTF for this initial proof-of-concept study, but a broader search of the parameter space for optimizing the method will be presented in a follow-up study).

**Figure 2:**
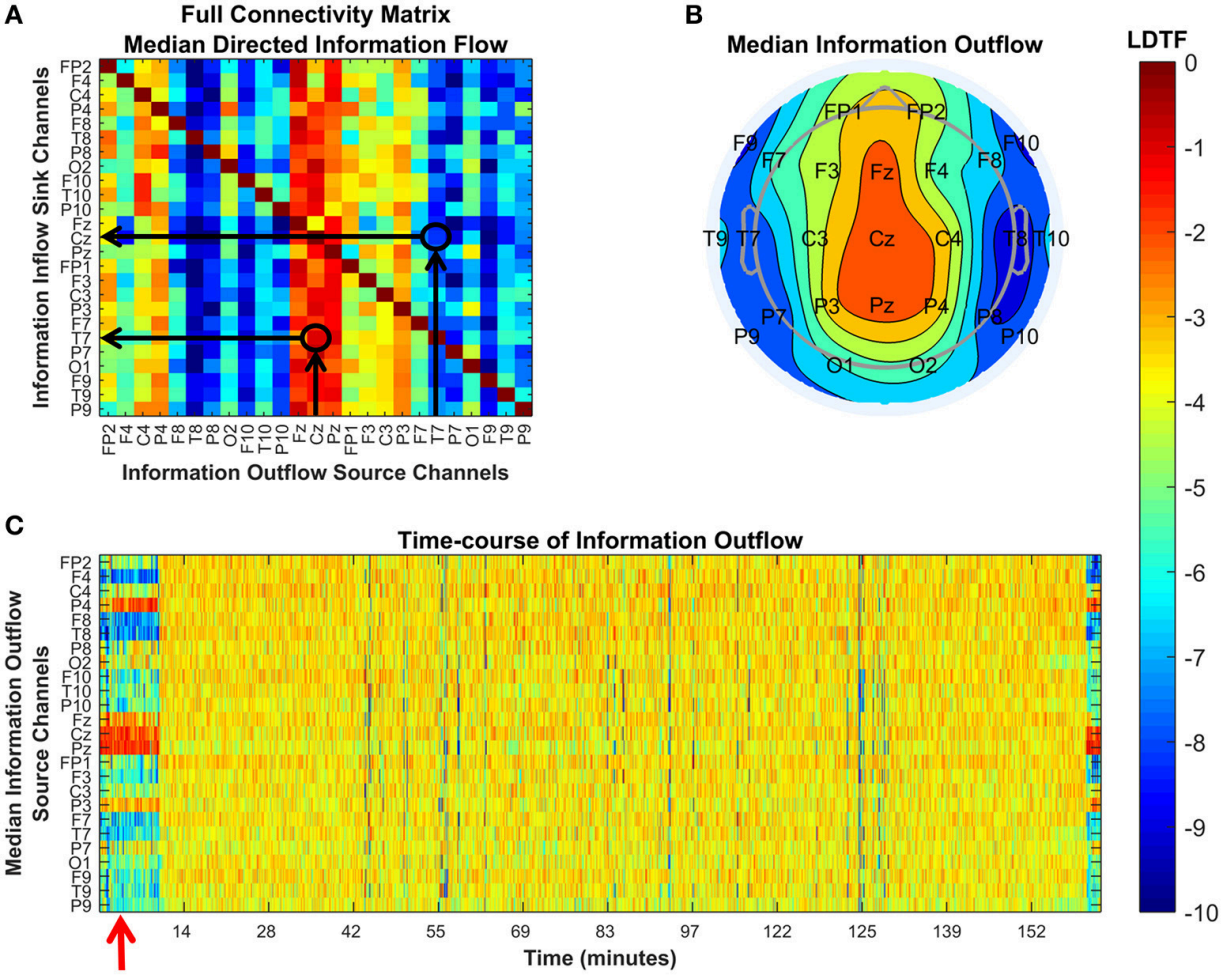
Explanations of the plots used to visualize the DTF analysis. Each panel shows different properties of the connectivity as quantified by the DTF in our analysis. **(A)** Visualization of the full connectivity matrix within a given frequency range (here 8–12 Hz) for a specific 1-s segment from a given patient (#6). Each element in this matrix represents the median LDTF from a given source channel (x-axis) to a given sink channel (y-axis), and the strength is given by the color (color bar, right). Two examples are indicated by black arrows and circles in the matrix, showing the relatively strong (red) information flow from Cz to T7, and the relatively weak (blue) information flow in the opposite direction, from T7 to Cz. **(B)** Topographical representation of the source channels' strengths of information outflow from the same 1-s segment presented in **(A)**. The positons of channel labels indicate their relative locations on the head as seen from above (nose pointing up). In this 1-s segment, the medial channels are strong, whilst more lateral channels are weaker, sources of information outflow. **(C)** Shows how each channel's strength of information outflow changes with time (x-axis). Red arrows mark important points. Here, it marks the time of the 1-s segment visualized in the figures in **(A,B)**. All figures use the same color scale, shown by the color bar.

We transformed the measure of information flow (in the DTF sense) by taking the logarithm of the DTF values (LDTF). This gave us the main metric that we used for comparing the states of the patients, LDTF_ij_(f) = log(DTF_ij_(f)). Here, DTF_ij_(f) denotes the sensor space “information flow” from electrode j to electrode i, at frequency f. Taking the median of these values over the frequency range of interest, yields a matrix representing the normalized directed “connectivity,” or “information flow,” between all electrodes within the given range of frequencies (Figure 2A). To investigate how the activity in each channel typically influenced the activity in all other channels, the matrix of LDTF values was compressed, yielding a 1 × 25 array of values quantifying the median “*information outflow*” from each channel (Figure 2B). Thus, the “information outflow” from channel *j* was calculated by taking the median of all LDTF_ij_-values directed from a channel *j* at time t, LDTF_j_(f) = median(LDTF_ij_(f)). Calculating the LDTF_j_(f) sequentially for all 1-s segments from a given patient, results in a time-course describing how the information outflow from each channel changes with a 1-s temporal resolution (Figure 2C).

Throughout this study, the median was used when a summary statistic was needed, because it is more robust than the mean with regards to outliers caused by artifacts in the EEG data (Schumacher et al., 2011; Dukic et al., 2017). This is particularly important since the data in this study was not manually inspected and cleaned.

### Classification algorithm

To assess whether the LDTF could distinguish between the anesthetized and awake state in patients, we needed a method for objectively classifying the data driven purely by the LDTF values. This was done by separating the data set into two parts: a learning set and a test set. The training set is was used to quantify the properties of the LDTF in the anesthetized and awake state for all except one patient in the data set. Then the generalizability of these differences was tested by their ability to correctly classify the state of the final patient every second.

More concretely, an in-house algorithm was implemented to objectively classify the patient as anesthetized or awake based on the LDTF values calculated from 1-s segments of raw EEG. To achieve this, a 5-s moving median LDTF was calculated for a patient, yielding a smoothed time-course of LDTF values with 1-s time resolution (the test set). The LDTF values from all other patients were pooled together (resampled to control for autocorrelation time in the LDTF and to make the number of data points in each condition of equal size), and were used to generate distributions of LDTF values for all channel pairs (the training set). So, when classifying the state of a given patient, the basis for the classification were the empirical distributions of LDTF values from all the other patients.

Since the anesthesiologist had marked the time points when each patient transitioned between anesthetized and awake states throughout the clinical procedure, we had a ground truth for the state if the patient at every time point. Each time point in the smoothed time-course of LDTF values in the test set was compared with the distributions of LDTF values from the awake and anesthetized state from the training set. If a patient's LDTF values for a given time-point were more likely to be drawn from the awake than anesthetized distribution, the time-point would be classified as awake. Otherwise it would be classified as anesthetized. Finally, this data-driven classification was compared with the anesthesiologist's clinical classification to indicate the accuracy of our algorithm.

The algorithm is explained in more detail below, and the implementation is visualized in pseudo-code in Textbox 1.

TEXTBOX 1 **Pseudocode for the classification algorithm.**

   for all_patients

      for all_other_patients

         for all_channel_pairs (ij)

            % generate training sets for classification

            pool all data from anesthesia into one variable

            pool all data from awake state into another

 

   % calculate statistics for each state

     calculate median of LDTF across anesthetized and awake state

     calculate standard deviation of LDTF for each states

   % making classification for current patient

    for all_one-second_segments (from fifth second)

        for all_channel_pairs (ij)

            calculate median LDF_ij across previous 5 seconds (mLDTF_ij(t))

 

   % given a matrix mLDTF(t) containing all mLDTF_ij(t) values

    calculate likelihood, P_state, of drawing **mLDTF(t)** from each state

     if P_awake > P_anesthetized

        classify as awake

   else

        classify as anesthetized

 

    calculate accuracy, sensitivity, and specificity of classification

As can be seen from the pseudo code, the classification algorithm was developed, implemented, and tested using a “leave one out”-cross validation approach. For each patient, the data from all other patients were pooled together into either “*awake*” (before or after anesthesia) or “*anesthetized*” (during stable anesthesia) categories. The pooled data of LDTF_ij_(f) values formed the training set that the state classification was based on. From this training set, the median of LDTF_ij_(f) over all time points within a given state (“awake” or “anesthetized”) was calculated, giving the matrix of median “information flow” between all EEG electrodes: **mLDTF**^state^ (as in Figure 2A). The standard deviation, ${\text{s}}_{\text{ij}}^{\text{state}}$, of the information flow within a given state was also calculated, for each channel of interest. These statistics were sufficient to define distributions of LDTF_ij_(f) values for all channel pairs, conditional on the patient state.

To classify the state of a patient at time *t*, we compared that patient's LDTF_ij_(f) values at time *t*, with the training set distributions generated from the other patients' data. To get a less noisy estimate of the test-patient's LDTF_ij_(f) values at time *t*, we calculated the median of LDTF_ij_(f) values across the preceding 5 s to give the matrix of test parameters, **mLDTF (t)**^test^. In other words, this matrix contains the median strength of information flow between all channel pairs of interest in the 5-s interval leading up to the current time, *t*. This can be understood as the typical LDTF_ij_(f) values in the 5 s leading up to the time *t*.

From the **mLDTF (t)**^test^ values, we could quantify the likelihood that the values arose from “awake” and “anesthetized” state distributions characterized by the training set described above. This was done by taking the product, across all channel pairs, of the probability that the observed **mLDTF (t)**^test^ values at a given time was drawn from the distributions of LDTF values observed from the other patients in the study. To calculate the relevant probabilities, we assumed that the distributions LDTF values for each channel pair was approximately Gaussian, which can be described by the empirical medians (mLDTF^state^) and standard deviations (s^state^). The formula for the likelihood that the **mLDTF (t)**^test^ values were drawn from “awake” and “anesthetized” state distributions then becomes the following:

$$\begin{array}{l} P\left(state|mLDTF{\left(t\right)}^{test}\right)\\ =\prod_{i\neq j}^{L}\frac{1}{s_{ij}^{state}\sqrt{2\pi }}exp\left(\frac{-{\left(LDTF_{ij}^{test}\left(t\right)-mLDTF_{ij}^{state}\right)}^{2}}{2*{\left(s_{ij}^{state}\right)}^{2}}\right) \end{array}$$

Here, **mLDTF (t)**^**test**^ is an L-by-L matrix containing all the information flow values from the test-patient at time, *t*, between the *L* channels of interest, and *state* is either “anesthetized” or “awake.” Each term in this product is just the probability of drawing a given value of $LDTF_{ij}^{test}\left(t\right)$, given a Gaussian distribution with mean $mLDTF_{ij}^{state}$ and standard deviation $s_{ij}^{state}$.

For each time step, P (*anesthetized* | **mLDTF (t)**^**test**^) was compared with P (*awake* | **mLDTF (t)**^**test**^), and the patient was classified based on the following rule:

$$PredictedState\left(t\right)=\{anesthetized,ifP\left(anesthetized|mLDTF{\left(t\right)}^{test}\right)>P\left(awake|mLDTF{\left(t\right)}^{test}\right)\;awake,otherwise$$

This means that the patient was classified as anesthetized at some time, t, if the corresponding LDTF^state^ values were most likely to be drawn from the observed distributions of LDTF values from other patients under anesthesia. In contrast, if the LDTF^state^ values at some time, *t*, were most likely to be drawn from the observed distributions of LDTF values from other patients in the awake state, the patient was classified as awake. The classifications were then compared to the reported clinical states, and this comparison was used as the basis for calculating the accuracy of the algorithm. Similarly, the algorithm's sensitivity and specificity of classification were calculated to avoid bias in the accuracy calculation due to the data set from anesthetized state being larger than that from the awake state.

### Statistical tests

We tested which, if any, LDTF_j_(f) values changed between the awake and anesthetized states by applying independent Wilcoxon rank-sum tests between conditions for each channel, with Bonferroni correction for multiple comparisons. First, the tests were done using all available data, before the tests were redone with downsampled data to make distributions from the wake and anesthetized states of equal size and controlling for autocorrelation in the LDTF values. The downsampling consisted of choosing LDTF values from every 100th 1-s segment to reduce autocorrelation in the data (increasing independence between data points), and further downsampling the anesthesia distribution (using only 1 of every 50 segments) to make the distributions of comparable size. Based on the *post-hoc* analyses of the LDTF distributions, the full analysis described above was redone starting from a reduced number of EEG channel, only including those channels that were found to be different between the wake and anesthetized states for the full model.

## Results

Ten patients (8 male, 2 female) were recruited to the study, but one of the recording files was lost due to technical failure of the recording station. One patient that fell asleep before the surgical anesthesia was started was also excluded, leaving us with eight adult patients (6 male, 2 female), with a median age of 59 years (range: 39–83 years), that were included in the analysis. From each of these patients, continuous 25 channel EEG was recorded throughout the surgical procedure (median duration: 3 h 15 min; range: 2 h 11 min−3 h 48 min). Each recording contained a section from anesthesia in which the patient was regarded as fully “anesthetized” (median: 2 h 43 min; range: 1 h 40 min−3 h 23 min), and shorter sections from before anesthesia (median: 6 min; range: 0 min−11 min) and after anesthesia (median: 3 min; range: 1 min−10 min) in which the patient was considered “awake.”

**Figure 3:**
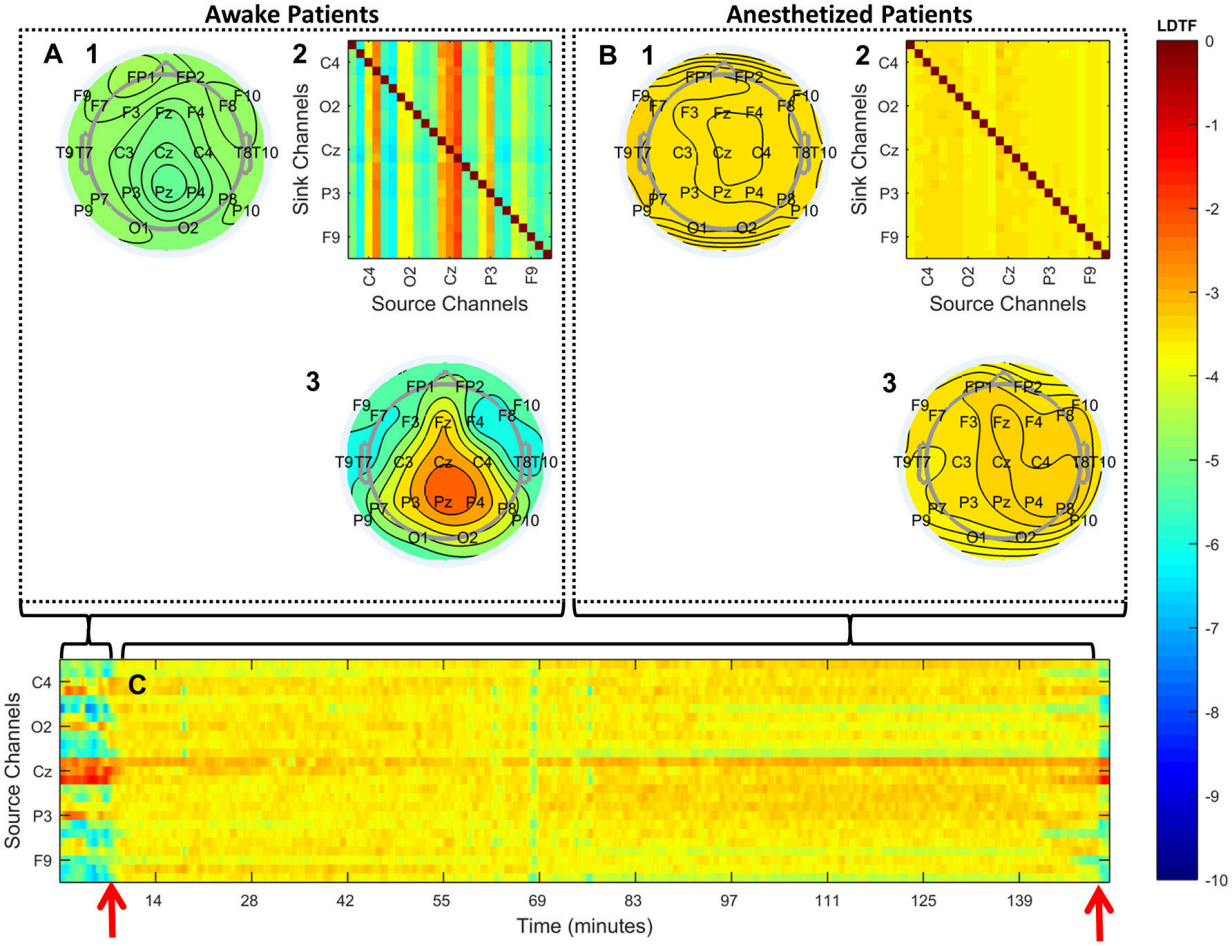
Qualitative summary of the DTF analysis. **(A,B)** Summarize the structure of median connectivity in the “awake” and “anesthetized” states respectively, given by LDTF, calculated from the data pooled from all patients. **(A2,B2)** Show the full directed connectivity matrices. Each element in the matrix quantifies the median information flow, mLDTFij, between the j'th source channel (x-axis) and the i'th sink channel (y-axis). The topographical plots show the distribution of information outflow and inflow regions (sources and sinks) on the scalp. **(A3,B3)** Depict the median information outflow from each point, while **(A1,B1)** depict the information inflow to each point, on the scalp. **(C)** Shows the time courses of information outflow from all channels for a single patient (#7), illustrating the dynamics of the measure over time. The time-course is smoothed using a moving median calculation, taking the previous five 1-s segments into consideration. The two red arrows indicate the time of loss of verbal contact and response (left) and the time point when the patient again responded verbally (right). Note that only a few of the channels in **(A2,B2,C)** are labeled because of space restrictions. See Figure 2 for the full set of channel labels. All figures show the median logarithm of DTF within the alpha frequency range. The color scale on the right indicates the LDTF values for all panels: red colors indicate strong, while blue colors indicate weak, information flow.

Analyzing the properties of the DTF calculated from these recordings revealed differences between the “anesthetized” and “awake” patients. Qualitatively, the overall pattern of “information outflow” changed from being heterogeneous in the “awake” state, to being more homogenous in the “anesthetized” state (Figures 3A,B). In particular, in the “awake” state, a relatively strong source of outflow was apparent in the posterior region (e.g., Pz in Figure 3A3), whereas this region appeared far less prominent in the “anesthetized” state (Figure 3B3). The differences in the DTF-derived properties were apparent for individual patients when investigating the sources of “information outflow” as a function of time (Figure 3C). The structure of “information outflow” changed with time, and it did so abruptly around the time points when the patient's state of anesthesia was reported to change. When the patient regained consciousness as assessed by the anesthesiologist, the pattern changed back, becoming qualitatively similar to before anesthesia.

**Figure 4:**
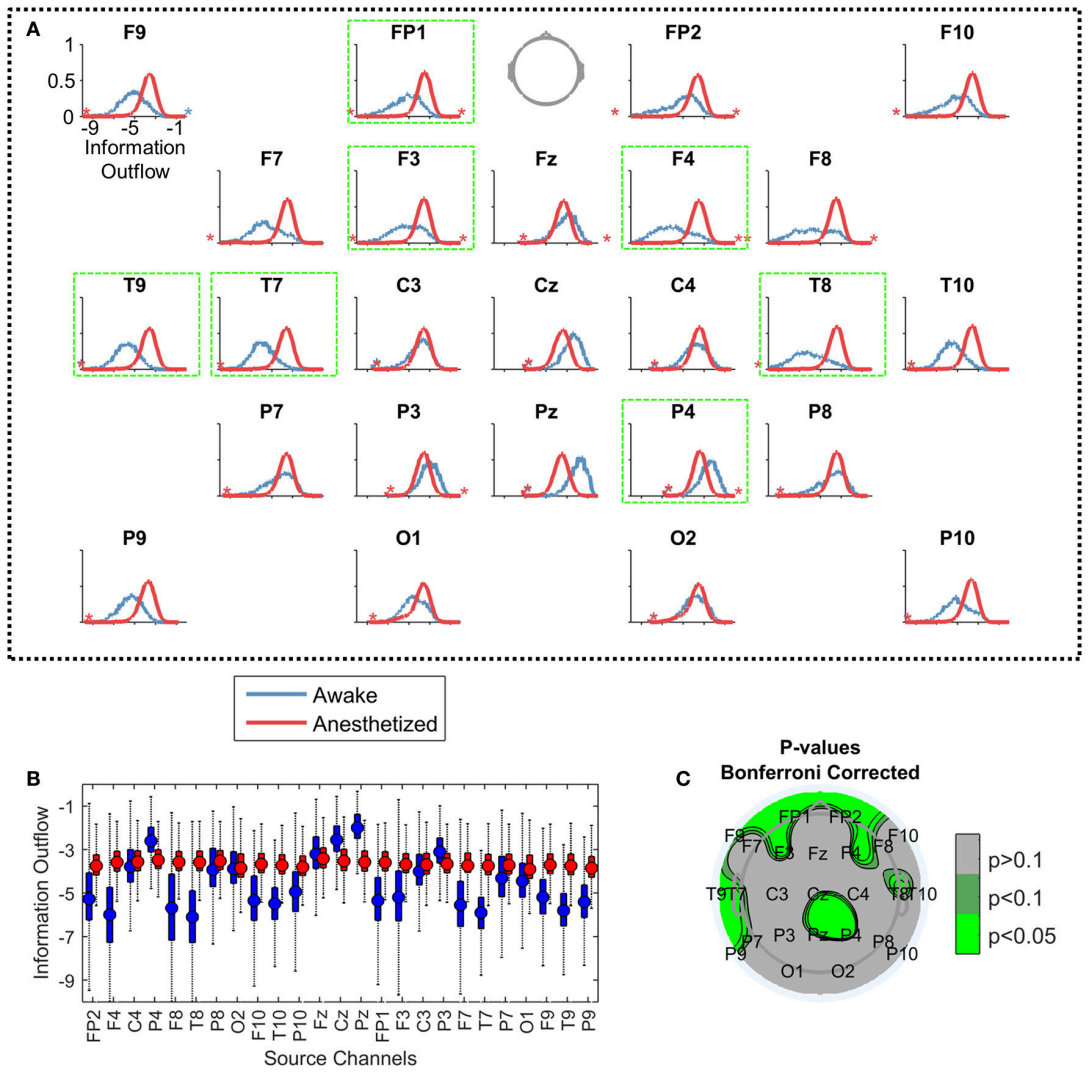
Comparing the distributions of information outflow from different EEG channels. Each plot in **(A)** visualizes the distributions of information outflow values from a single channel, from the “awake” (blue) and “anesthetized” (red) states. Again, the data is pooled from all patients in the given state. The vertical positions of the stars indicate the proportion of outliers on the indicated sides of the distributions. The axes are equal in all panels [shown only for F9, upper left in **(A)**], with the y-axis showing the probability density of observations (range: 0 to 1), and the x-axis indicating the LDTF value (range: −10 to 0). The panels are ordered topographically as if the head is seen from above with the nose pointing up. The panels with a bright green frame mark the channels with significantly different distributions between the “awake” and “anesthetized” states. In **(B)**, the same information is given as boxplots with the same color coding. The mean of each distribution is given by a filled circle in each box, and the whiskers indicate the standard deviation of the distributions. Outliers have been omitted. The y-axis shows the information outflow, and corresponds to the x-axes of the plots in **(A)**. **(C)** Shows a topographical plot of the results from the statistical test comparing the distributions of LDTF information outflow values for each channel between awake and anesthetized states—controlling for autocorrelation in the information outflow, resampling to make the data sizes similar, and correcting for multiple comparisons. Green colors indicate regions with significantly different median information outflow distributions in awake and anesthetized states.

Even though the main patterns of the LDTF values were quite stable within states, they were not static, but varied within state specific distributions (Figures 4A,B). The distributions of “information outflow” in the “anesthetized” state looked qualitatively quite similar for each channel, while distributions in the “awake” state seemed to depend more strongly on the scalp position. Indeed, all channels in the awake state were found to be significantly different from the “anesthetized” state distributions (*p* < 0.01; Wilcoxon rank-sum tests with Bonferroni correction for multiple comparisons). Also after controlling for autocorrelation time in the measure (~100 s), and resampling to make the populations of similar size (subsampling of anesthesia data to the size of awake data), the distributions of 8 channels remained significantly different with *p* < 0.05 using the same statistical test (F4, P4, T8, Pz, FP1, F3, T7, and T9; see Figure 4C).

**Figure 5:**
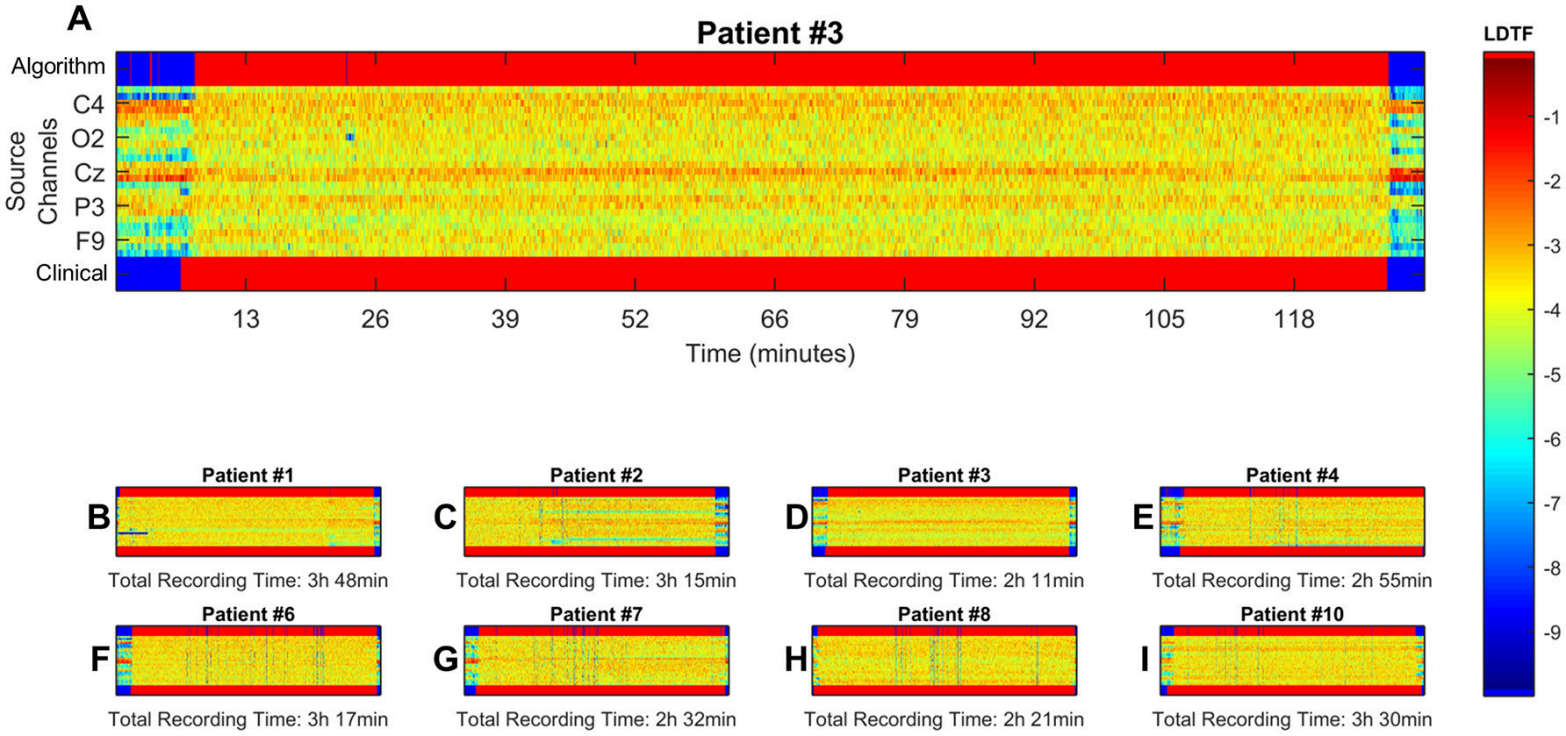
Visualization of results from classification algorithm. Here, data from all channels with significantly different distributions of median information outflow between states were used for continuous classification the patient state. (**B–I**, zoomed example in **A**). Show the time course of the LDTF information outflow channels for each patient, the patient's state as reported by clinical staff, and the classification made by the algorithm. The middle region of each panel, containing the LDTF values for every channel, follows the color scheme defined in the color bar. The top and bottom bars show the corresponding conscious state of the patient as suggested by the algorithm and reported by the clinical staff respectively, and have their own color scheme: blue means “awake” while red means “anesthetized.” **(A)** Shows the same information as **(D)**, just enlarged to give a better impression of the dynamics, and numerated axes. Possible misclassifications can be seen as blue points or regions in the upper bars of each panel where one would expect a red color according to the clinical report (i.e., false positives: predicting “awake” when clinicians report “anesthetized”), and red regions where the clinical report suggest a blue color (false negatives: predicting anesthetized where clinicians report “awake”).

The distributions of LDTF_ij_ values could be used to classify the state of the patient (i.e., anesthetized vs. awake) solely based on the EEG data, as described in the section Classification Algorithm. The clinically observed changes in state were accompanied by abrupt changes in LDTF_j_(f) values, and the data-driven classification produced by our algorithm responded rapidly and corresponded closely to the state indicated by the anesthesiologist (Figures 5 B–I).

The accuracy, sensitivity, and specificity of the classification algorithm are shown in Table 1. These scores depended on whether the input data came from (1) all recorded EEG channels, or (2) only the channels with significantly different information outflow in the two states.

**Table 1:** Summary of predictive values for the classification algorithm.

**Channels**	**Accuracy**	**Sensitivity**	**Specificity**
All	0.9591	0.9587	0.9591
Significant	0.9579	0.8738	0.9639

## Discussion

We found significant differences in the properties of the DTF calculated from 1-s segments of raw EEG when comparing data from patients in the anesthetized state with data from the same patients when they were awake before or after the anesthesia. The observed differences could be used to objectively classify the state of single patients as “awake” or “anesthetized,” with a high degree of accuracy and high time resolution before, during, and after the anesthetic-induced unconsciousness.

### DTF-derived properties changed abruptly when propofol-induced loss of consciousness was reported

The properties of the DTF calculated from 1-s segments of raw EEG changed abruptly when consciousness was lost, but rapidly returned to the wake-like pattern when the patient woke up and regained communication. The heterogeneous pattern of “information outflow” observed in the “awake” patients changed to a much more homogenous pattern during anesthesia.

It is widely recognized that the concept of consciousness encompasses at least two dimensions: the level (i.e., arousal or wakefulness), and the content of consciousness (Laureys, 2005). In this study, we focused on the level of consciousness and the related behavioral responsiveness, which are obviously affected by general anesthesia and can readily be assessed by clinical examination. Thus, the patients were considered to be unconscious when they were behaviorally unresponsive as judged by the anesthesiologist. However, the intrinsic subjective state of the patient was not measured or controlled for, leaving open the possibility that some patients had dreamlike experiences during the surgery (Eer et al., 2009).

At least four previous studies have investigated how DTF is affected by changing states of consciousness (Kaminski et al., 2001; Gennaro et al., 2004; Bertini et al., 2009; Höller et al., 2014). Höller et al. (2014) tested DTF as a part of a larger study to find the measures best suited to classify patients with DOC at the group level. They found that differences in DTF, together with partial coherence, were the strongest classifiers for the separating vegetative and minimally conscious patients. Earlier, Bertini et al. (2009) investigated how the interhemispheric connectivity changed as a function of sleep stages and found that apparent “connectivity” quantified by DTF changed between wakefulness and stage 2 sleep. The change was particularly clear in the alpha frequency “connectivity” between the parietal electrodes P3 and P4, which are close to the region where we found clear changes from strong to weaker information outflow when going from awake to anesthetized state (Figure 2). Gennaro et al. (2004) showed that the DTF changed just after sleep onset. In particular, they found that “connectivity” from posterior electrodes to frontal electrodes was reduced upon falling asleep. Finally, Kaminski et al. (2001) also observed similar changes in DTF structure at the onset of sleep, specifically a “diminishing role of the posterior sources and an increasing effect of the anterior areas.” These findings, together with indications that DTF can be used to separate “vegetative” from minimally conscious and awake states (Höller et al., 2014), suggest that such changes in DTF may capture properties related to loss of consciousness in general, not just specific features of propofol anesthesia.

In addition to the DTF-studies, there is a vast literature concerning potential EEG properties related to changes in level of consciousness (Kreuzer, 2017). A comprehensive discussion of how our results compare to other potential measures for monitoring level of consciousness during anesthesia is beyond the scope of the first proof-of-concept study presented here. Furthermore, the fact that of our preprocessing steps excluded standard data cleaning procedures (such as spatial or temporal filtering, artifact rejection, resampling etc.) complicates comparisons with previous publications. That said, some points of interest in relation to previous literature should be noted. First, our finding of clear changes in the alpha band, especially in the frontal and lateral regions, is reminiscent of typical changes occipital and frontal alpha oscillations reported to be EEG signatures of loss and recovery of consciousness from propofol (Purdon et al., 2013). Secondly, we observed large changes in apparent information flow from frontal and posterior areas between wakefulness and anesthesia, while the asymmetry in information sources seemed to shift. This can be seen as support for the claim that anesthesia-induced loss of consciousness is related to a depression or functional disconnection of lateral frontoparietal networks (Hudetz and Mashour, 2016). Furthermore, it supports the general idea that connectivity may be crucial for determining the level of consciousness (Alkire, 2008; Alkire et al., 2008; Ferrarelli et al., 2010; Hudetz, 2012; Casali et al., 2013; Höller et al., 2014). Finally, the strong source of information flow observed near medial posterior channels in the awake state is reminiscent the so-called posterior “hot zone” referred to in a recent review about neural correlates of consciousness (Koch et al., 2016).

In sum, the method implemented here applied an effective connectivity approach in a frequency band that is consistently reported to be subject to change upon loss of consciousness. The results of our analysis seem, to the extent the comparison is valid, comparable to previously reported changes observed upon loss of consciousness. Importantly, DTF variables are calculated from short segments of EEG, and the observed patterns were quite stable within conditions and changed abruptly when conditions changed. These properties bode well for DTF, implemented in the way suggested here, being useful as a clinical marker of propofol-induced unconsciousness. However, further validation under different conditions is required for more general claims, and a thorough investigation of the parameter space and preprocessing steps should be conducted in order optimize the method for accuracy in classification.

### Potentially a clinically viable monitor of anesthesia

In this study we used DTF-derived measures calculated from raw data, without any form of preprocessing such as artifact removal or corrections, in order to approach the requirements of a clinically viable monitor of anesthesia. Using this method, the state of individual patients could be objectively classified as “anesthetized” or “awake” with high time resolution (1 s), accuracy, sensitivity, and specificity.

Even though the classification scores could possibly be improved by using preprocessed data, it is a requirement that no manual pre- or post-processing is necessary, to be used as the basis for a clinical monitor. In a real clinical setting, a monitor will not be used if it requires a lot of extra effort on the clinician's side. In particular, setting up a large number of EEG channels for every patient undergoing anesthesia is not practical. We showed that reducing the number of channels from the original 25 EEG channels recorded still reaches relatively high classification scores. However, the sensitivity suffered, and the method should be further optimized for lower numbers of electrodes if it is to become a valuable addition to the clinician's toolkit for monitoring anesthesia.

There are already commercially available EEG-based monitors of general anesthesia such as *Bispectral Index* (BIS), Cerebral State Monitor, *E-Entropy, SedLine*, and *Narcotrend* (Musialowicz and Lahtinen, 2014). These are often used to reduce the risk of unintended awareness during general anesthesia, and their use is recommended in the most recent Cochrane review (Punjasawadwong et al., 2014). Especially BIS is widely used, but although it is the most thoroughly studied of the available monitors, its reliability and validity for monitoring awareness has been questioned and extensively discussed in the literature over the last two decades (Myles et al., 2004; Avidan et al., 2008; Schnakers et al., 2008; Mashour et al., 2012; Goddard and Smith, 2013). Despite the recommendations, there have been several cases where patients, after waking up, reported awareness, explicit memories, and pain during BIS-monitored anesthesia, even when the monitor indicated deep levels of anesthesia (Mychaskiw et al., 2001; Schneider et al., 2002; Vuyk et al., 2004; Avidan et al., 2008).

The algorithm described here, made a new classification every second, based on the previous 5 s of data, giving it a very high temporal resolution. This beats the speed of some currently available methods by up to an order of magnitude according to simulation studies (Pilge et al., 2006). Depending on how large the changes in EEG-patterns were, and how fast they were presented, the tested monitors (BIS, Narcotrend, and Cerebral State Monitor) responded with time delays between 14 and 155 s. Together, the fast response of the method presented here and its lack of pre-processing steps give reason to believe this method may form a basis for a clinically viable monitor of anesthesia.

However, it should be noted, that even though the classification accuracy reported here are high, the dataset is much too small to say whether the proposed method has the capacity to detect extremely rare but relevant events such as unintended awakenings (Sebel et al., 2004; Ghoneim et al., 2009). Furthermore, the patients included in this study did not undergo extensive interviews regarding whether they experienced anything during the surgery or not. Therefore, we do not know for a fact that all patients remained unconscious throughout the surgical procedure, although none of the patients spontaneously reported an unintended awakening. For future studies assessing methods for monitoring of conscious state, this should be improved, and the patient's own report of experience should be taken as the ground truth for classification.

### Methodological considerations

Our choice of focusing on the alpha band was empirically driven and based on preliminary analyses (see Figure S1). In addition, the alpha range has repeatedly been reported to change between presumably conscious and unconscious states in several relevant studies (Kaminski et al., 1995; Gennaro et al., 2004; Bertini et al., 2009; Höller et al., 2014). There, the alpha band was reported to show large changes between conscious and unconscious states, in anesthesia as well as sleep and disorders of consciousness.

Pre-processing steps such as filtering and artifact rejection were omitted deliberately in this study, since one of the main goals was to test whether DTF could potentially be used in a clinical setting for robust, real time, classification of anesthetized and wake states, from clinically relevant EEG data (for results from tests investigating the effects of typical preprocessing steps, and other methodological considerations, please see the Supplementary Material). In particular, we used 1-s segments of raw EEG to calculate the DTF based measures, in order to investigate whether there are changes that can be captured by the DTF calculated from such brief segments of raw EEG. If we could find changes in the DTF properties calculated from such short segments, this would suggest that DTF could be a practical method for monitoring state of anesthesia in clinical medicine.

Some authors have argued that the DTF should be calculated from long segments of EEG to give a reliable estimates of connectivity (Kaminski et al., 2001). However, due to the non-stationarity of EEG, the use of too long segments may cause an estimate of the connectivity to reflect a mixture of states. In addition, the reliability of estimation also depends on the EEG being devoid of artifacts, as noise in the signal may distort the estimates of connectivity by the DTF. Since we intentionally avoided preprocessing steps for cleaning data, we could not be sure that long segments of EEG would be free from artifacts, and needed another way to reduce the number of artifacts. We chose to consistently use short segments of EEG, and take the median across several segments within conditions to obtain estimates of the connectivity within that condition. As was mentioned earlier, the median was chosen due to its robustness to outliers, and this approach has been shown to be comparable to manual cleaning of artifacts in previous work (Schumacher et al., 2011; Dukic et al., 2017).

The DTF has been applied to electrophysiological data with a wide variety of model orders, segment lengths (ranging from sub-second to several minutes duration), and preprocessing steps (Kaminski and Blinowska, 1991, 2014; Ding et al., 2000; Kaminski et al., 2001; Gennaro et al., 2005; Bertini et al., 2009; Schumacher et al., 2015). That said, our choice of parameters in the analysis may have had an impact on the precision of inference about the underlying brain connectivity (Florin et al., 2011; Kaminski and Blinowska, 2014). However, whether the scalp level connectivity estimates give good estimates on the underlying, neural connectivity is in any case disputed (Brunner et al., 2016; Kaminski and Blinowska, 2017). In addition, to reiterate, the aim of this study was to investigate whether the anesthetized state could be objectively separated from the awake state in humans based on properties calculated from spontaneous, raw scalp EEG, not to estimate underlying brain connectivity. For this purpose it is of little importance whether or not the obtained results reflect neural connectivity.

## Conclusions

We have shown that properties of the DTF calculated from 1-s segments of raw EEG changed abruptly as patients undergoing surgical propofol anesthesia with remifentanil lost and regained wakefulness and behavioral responsiveness as judged by trained clinical staff. These changes could be used to reliably and precisely distinguish anesthetized from awake state in individual patients with a high temporal resolution. These results were achieved using raw EEG data from as little as eight electrodes, demonstrating the possible clinical viability of this approach. Therefore, we propose that data-driven classification algorithms based on properties of the DTF calculated from 1-s segments of raw EEG may become useful for developing future tools for objective, real-time monitoring of the conscious state in patients undergoing anesthesia.

## Author contributions

PGL was involved in conceptualization and design of the study, data acquisition, analysis and interpretation of data, and critically revised the manuscript. JFS was involved in conceptualization and design of the study, analysis and interpretation of data, writing and critical revision of the manuscript. FK was involved in the surgery, and contributed to and approved the manuscript. LR was involved in conceptualization and design of the study, data acquisition, and critically revised the manuscript. BEJ performed most of the data analysis and writing of the manuscript, and was involved interpretation of data and critically revised the manuscript.

### Conflict of interest statement

The authors declare that the research was conducted in the absence of any commercial or financial relationships that could be construed as a potential conflict of interest.
