# Supplementary material for: Distinguishing Anesthetized from Awake State in Patients: A New Approach Using One Second Segments of Raw EEG
Source: Front Hum Neurosci. 2018 Feb 20;12:40. doi: 10.3389/fnhum.2018.00040 (PMC5826260; doi:10.3389/fnhum.2018.00040)
Supplement: Supplementary file 1 [file DataSheet1.DOCX]

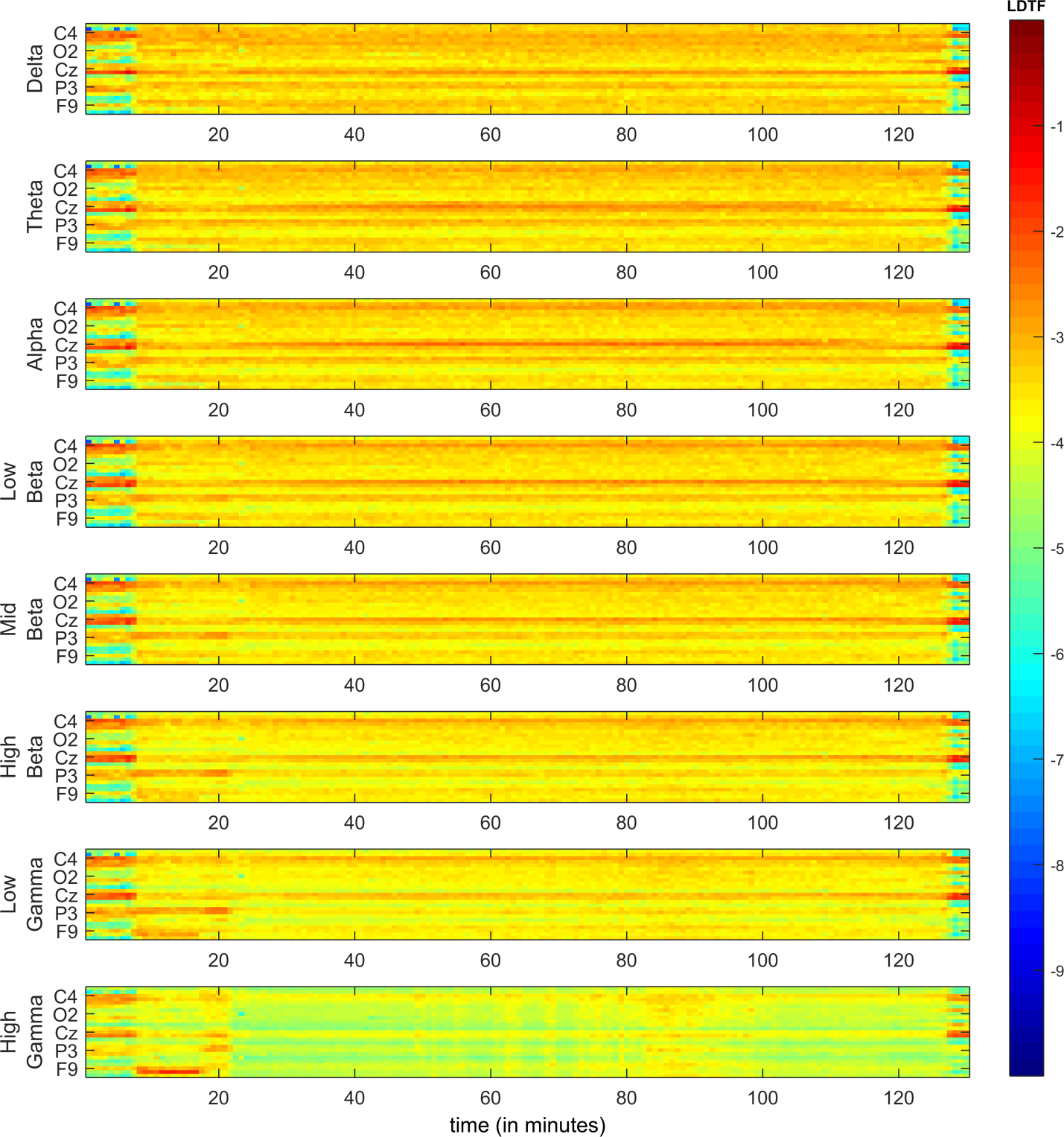
**Fig S1: Time-courses of information outflow sources for several frequency bands – Patient #3.** These panels indicate the changes in the information outflow for a range of different frequency bands. The data from all patients was analyzed in the delta (0-4 Hz), near theta (5-9 Hz), near alpha (10-15 Hz), low beta (16-20 Hz), mid beta (21-25 Hz), high beta (26-31 Hz), low gamma (32-37 Hz), and high gamma (71-75 Hz) frequency bands, and the results were assessed qualitatively to focus the choice of parameters to a given frequency band for the remaining analysis. Results for patient #3 are used here as an example. The color bar indicates the source strengths in the same way as in all other figures.

## Possible confounding factors

It is well known that EMG signals from nearby muscles can significantly contaminate EEG signals ^48^, particularly in the peripheral leads. Propofol partially blocks muscle activity ^49^, and changes in EMG might therefore be a cause of the observed differences between the states investigated here. However, there are reasons to believe that EMG did not significantly contaminate our main findings.

First, a single dose of the neuromuscular blocker cisatracurium was given at the onset of propofol anesthesia, which was sufficient to fully block all muscle activity for 10-20 minutes, followed by a partial recovery (still in the presence of propofol for more than one hour). However, this transient (10-20 min) full block of muscle tone did not produce any detectable changes in DTF (see for example Fig 4). Secondly, since our DTF analysis was based on a narrow frequency band (8-13 Hz), whereas propofol is known to induce EMG changes above 20Hz ^49^, propofol should have virtually no effect within the frequency band used in our study (see Fig S1).

Another factor that changed between states in our experiments was whether the patient had the eyes open. In general, eyes were closed during propofol anesthesia, but kept open during the periods of wakefulness before and after surgery. To check if this could affect assessment of connectivity, we performed *post hoc* experiments in volunteers to measure effects of eye opening and closing in the wake state. However, we found no significant differences between the states (see Supplementary figure S2-4).


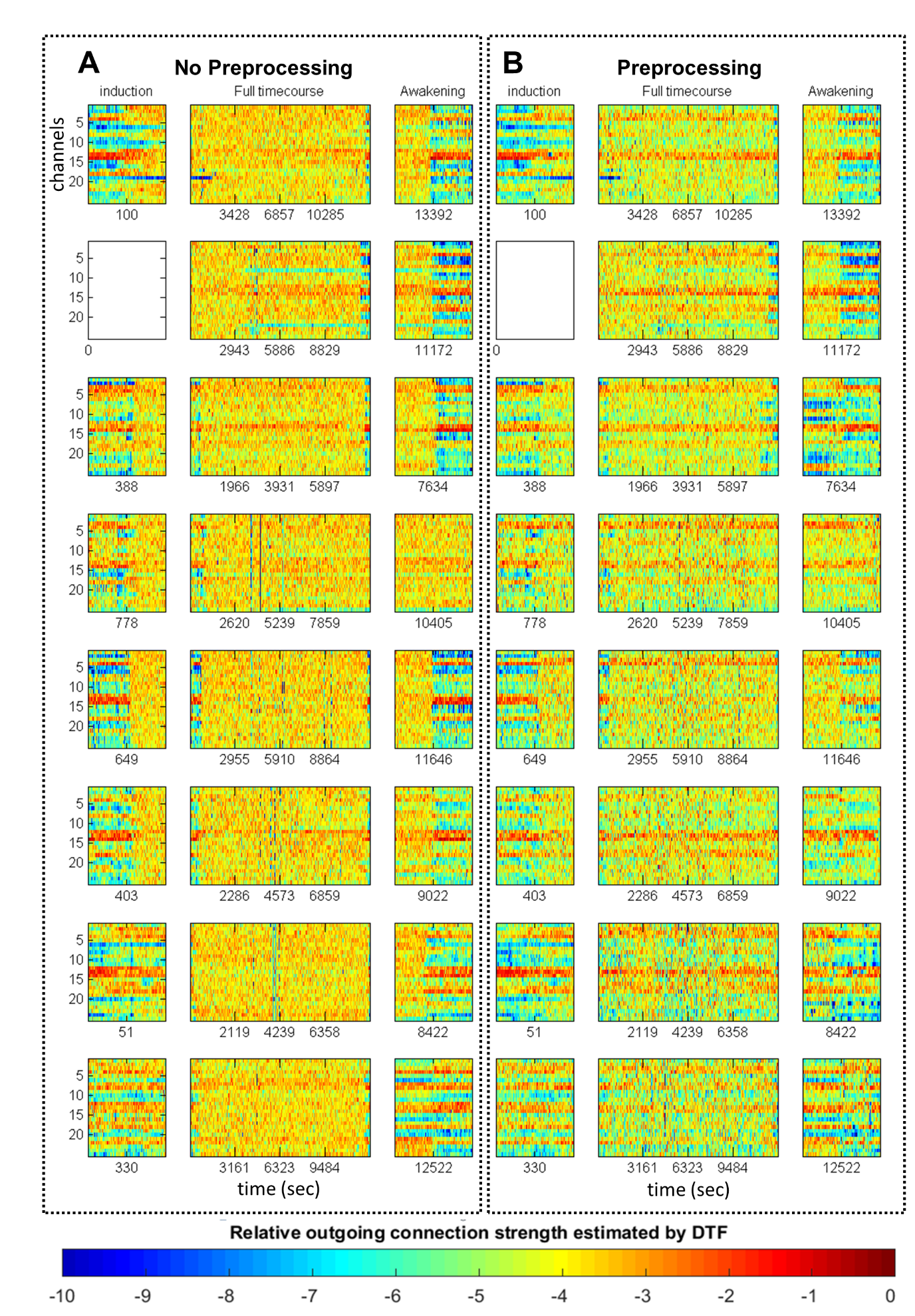


**Fig S2: Comparing results with and without simple preprocessing of the EEG data.** Panel A show results (outgoing connectivity) from the DTF analysis procedure used in this without preprocessing of the data by filtering, artefact rejection, or subtraction of means study (i.e. the same procedure as used in the main Figures 1-4). Panel B show the corresponding results from DTF analysis after preprocessing of the data: filtering with 1Hz high pass, and 40Hz low pass cutoff frequencies, using the forward and backward filtfilt procedure in Matlab, and subtraction of means in each 1 second segment. In panels A and B, results from the same 8 patients (rows) are shown side by side. For each patient, 3 plots are shown in each panel, A and B. The middle plot shows the full time course. The left and right plots show, at an expanded time scale, the transitions from wake to anesthesia (Induction) and from anesthesia to waking up (Awakening), respectively, to emphasize these important transitions. In the second row, the first plot (Induction) is blank because no EEG was recorded during the transitions from wake to anesthesia in this patient. The numbers on the time axes mark the time (in seconds) from the beginning of EEG recording. The numbers below each left (Induction) and right (Awakening) hand plot indicate the times when the clinicians indicated that the patient became unconscious and regained consciousness, respectively. The color bar indicates the color scale for strength of outgoing connectivity.


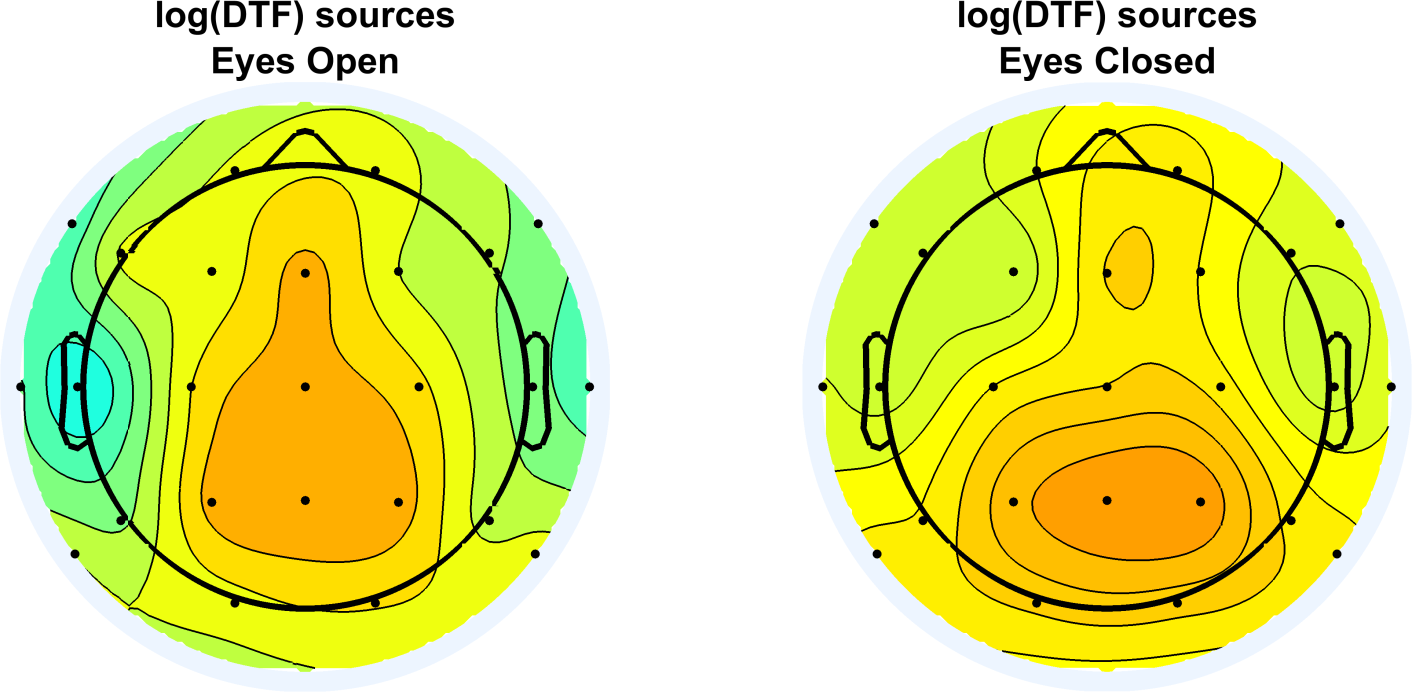


**Fig S3: Information outflow topographies with eyes open and eyes closed.** Post hoc experiments were done to compare the DTF information outflow when subjects have their eyes open or closed. Five two minute segments of EEG was recorded with a 64-channel EasyCap and BrainProducts amplifiers for each condition (eyes open and eyes closed). However, we only considered the channels most closely corresponding to the channels used in the data collection for the data analyzed in the main article, to make the data as comparable as possible. The DTF was then calculated in the same way as the for the data presented in the main article. The figures show the information outflow pooled across all recordings within each condition. Both topographies show the more heterogenous information outflow (stronger medial and posterior regions) seen in the wake state. See Fig S3 for statistical comparison.


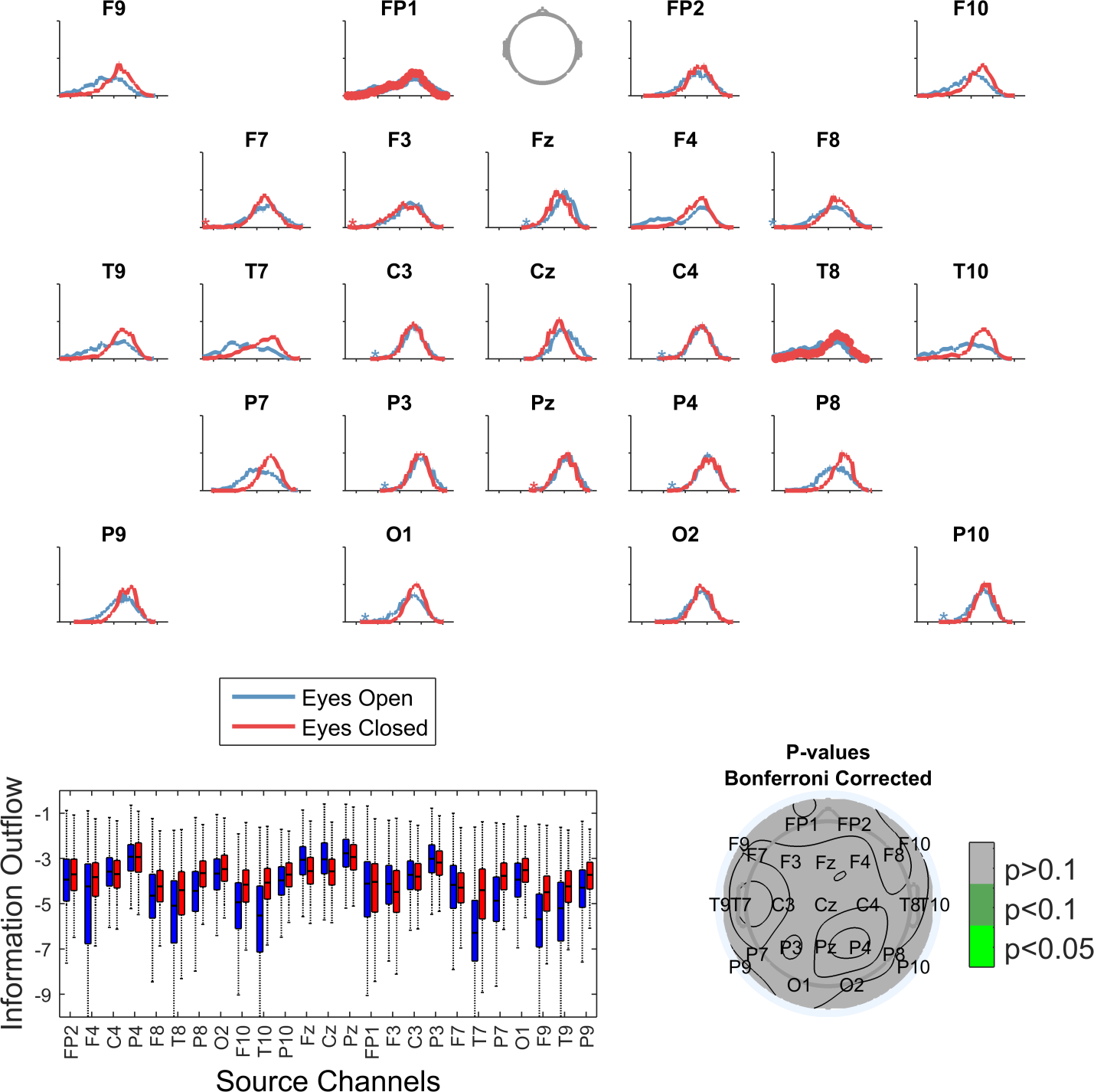


**Fig S4: Statistical comparison of the information outflow across the scalp with eyes open or closed.** The upper panels show the distributions of all information outflow values for all 25 channels analyzed from the post hoc experiments. Blue and red lines outline the distributions from the ‘eyes open’ and ‘eyes closed’ conditions, respectively. In the bottom left panel, the same information is visualized with boxplots. Finally, in the bottom right corner, a topographical plot of the P-values from a Bonferroni corrected comparison of the distributions. Qualitatively, no channel has statistically different distributions when comparing the ‘eyes open’ and ‘eyes closed’ conditions. Qualitatively, the distributions arguably look more similar to ‘awake’ distributions than those seen in the ‘anesthetized’ states (see Fig 3).


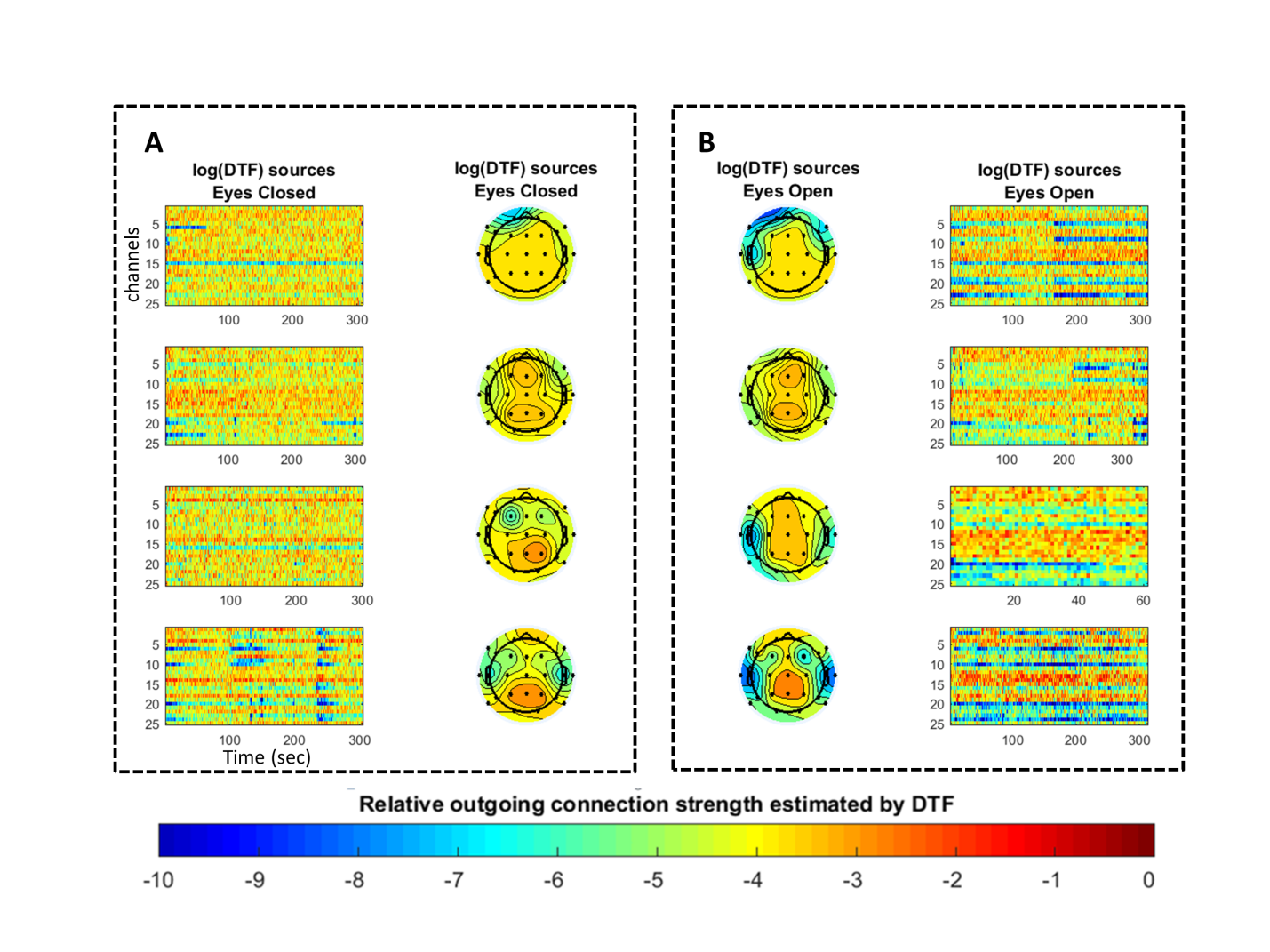


**Fig S5: Individual time courses and topographical source strength maps of healthy volunteers** **(controls)** **with eyes open and eyes closed.** Figures in panel A show results from controls with eyes closed, while the figures in panel B show results from the same individuals with their eyes open. Each row contains the resulting DTF connectivities (both a time course and a topographical map of the median outgoing connection strengths from that individual) from our control experiments, comparing eyes open and eyes closed states in four healthy, awake individuals (other than those included in the main study). These recordings were done with a different EEG-system with a different type, number, and montage of electrodes (BrainAmp DC, 64 channels, 5kHz, 0-1000Hz filters in acquisition). The resulting maps of outgoing connection strengths seem less stable than in the main results of the manuscript, but showed no statistically significant difference between states (please see supplementary figure S3 for a statistical comparison).
